# Genome-Wide Binding Patterns of Thyroid Hormone Receptor Beta

**DOI:** 10.1371/journal.pone.0081186

**Published:** 2014-02-18

**Authors:** Stephen Ayers, Michal Piotr Switnicki, Anusha Angajala, Jan Lammel, Anithachristy S. Arumanayagam, Paul Webb

**Affiliations:** 1 The Methodist Hospital Research Institute, Genomic Medicine Program, Houston, Texas, United States of America; 2 Tecnologico de Monterrey School of Medicine, Monterrey, NL, Mexico; Univeristy of California Riverside, United States of America

## Abstract

Thyroid hormone (TH) receptors (TRs) play central roles in metabolism and are major targets for pharmaceutical intervention. Presently, however, there is limited information about genome wide localizations of TR binding sites. Thus, complexities of TR genomic distribution and links between TRβ binding events and gene regulation are not fully appreciated. Here, we employ a BioChIP approach to capture TR genome-wide binding events in a liver cell line (HepG2). Like other NRs, TRβ appears widely distributed throughout the genome. Nevertheless, there is striking enrichment of TRβ binding sites immediately 5′ and 3′ of transcribed genes and TRβ can be detected near 50% of T3 induced genes. In contrast, no significant enrichment of TRβ is seen at negatively regulated genes or genes that respond to unliganded TRs in this system. Canonical TRE half-sites are present in more than 90% of TRβ peaks and classical TREs are also greatly enriched, but individual TRE organization appears highly variable with diverse half-site orientation and spacing. There is also significant enrichment of binding sites for TR associated transcription factors, including AP-1 and CTCF, near TR peaks. We conclude that T3-dependent gene induction commonly involves proximal TRβ binding events but that far-distant binding events are needed for T3 induction of some genes and that distinct, indirect, mechanisms are often at play in negative regulation and unliganded TR actions. Better understanding of genomic context of TR binding sites will help us determine why TR regulates genes in different ways and determine possibilities for selective modulation of TR action.

## Introduction

Previous unbiased studies of the genomic distributions of nuclear hormone receptors (NRs) have revealed several unexpected aspects of their mechanisms of action [1]–[9] Early models suggested that NRs bind DNA response elements (REs) that are commonly located within target gene promoters. From these positions, NRs modulate nearby gene expression via hormone-dependent changes in coregulator binding that, in turn, lead to changes in chromatin organization, histone modification and RNA polymerase II recruitment and processivity [10], [11]. ChIP studies, however, have revealed that: i) cognate NR REs are widely distributed throughout the genome and often far from obvious target genes, ii) ligand-dependent NR/DNA binding activities that were not seen *in vitro* can emerge in vivo and iii) NRs can display unexpected DNA binding preferences. Additionally genome-wide studies have confirmed suggestions that NRs can sometimes be recruited to target genes via interactions with heterologous transcription factors (TFs) and also revealed that NRs cooperate with unique subsets of heterologous TFs in composite modules, recognized through proximity of their DNA binding sites [5], [7], [12], [13].

Currently, there are no comprehensive genome wide studies of thyroid hormone (TH) receptor (TR) binding sites in liver cells and few in other cell types. It is therefore likely that complexities of TR genomic distribution are not fully appreciated. TH acts through two closely related receptors (TRα and TRβ) which are both members of the NR family, to regulate genes involved in cell growth and differentiation, homeostasis and energy metabolism [14], [15]. Both TRs commonly bind to specific TH response elements (TREs) within target gene 5′ proximal [16]. Nevertheless, there are many examples in which TREs are located within, or downstream of, their T3 regulated target genes [17], [18]. Further, a ChIP-on-chip analysis that interrogated TRβ binding events in the mouse cerebellum at locations from −8 KB/+2 Kb of known transcriptional start sites (TSS) revealed that 40% of 91 detectable TR binding events were in introns and not the proximal promoter [7]. Finally, a recent study, of similar design to one the described here, analyzed genomic localization of exogenously expressed TRs in a mouse neural cell line and showed that many genes that are induced by T3 lack a proximal TR binding site [19]. Thus, it seems likely that TREs are not restricted to proximal promoters and unbiased analysis of TR binding events is needed to define the extent of TRE locations and relationships to T3 regulated genes.

Although predominant models of TR action suggest that they act similarly to other NRs, subtle differences in the nature of gene regulation by TR can only be addressed by a non-biased, genome-wide analysis. It is thought that TRs interact constitutively with DNA as heterodimers with retinoid X receptors (RXRs) and this lack of ligand dependency has been confirmed for many individual [20], [21]. There are, however, suggestions that TRs are released from subsets of response elements upon hormone binding [22], based on studies of TR DNA binding preferences *in vitro* and investigation of TR/DNA interactions within promoters of negatively regulated genes. Neither idea has been tested at the genome level.

Although many TREs have been characterized, the defining components of TRE sequences have never been fully resolved. Canonical TREs that have been identified by conventional approaches are composed of single or multiple degenerate direct repeats of the AGGTCA half site spaced by four base pairs (DR-4) and comparison of these elements allowed establishment of a TRE consensus [23]–[27]. Analysis of individual TRE sequences has nevertheless also revealed significant individual variations in half site sequence and spacing and atypical half-site orientations, including everted and inverted palindromic repeats (ERs and IRs) [28] and there are suggestions that half site-proximal sequences may influence affinity for TRs versus other NRs that recognize AGGTCA half sites [29]–[32]. Further, TR-mediated transrepression of thyroid stimulating hormone, thyrotropin releasing hormone and superoxide dismutase involve distinct elements that do not resemble [33]–[36].

TRs are known to interact with several heterologous TFs, but the extent of TR-TF interactions and identities of these proteins are poorly understood. TRs bind AP-1 [37], [38], CREB [39] and p53 [40], [41] and also form composite elements with the chromatin boundary defining protein CCCTC binding factor (CTCF) [42]; recent computational analysis suggested up to 18% occurrence of TREs proximal to CTCF binding sites [43]. While these TFs and others may play important roles in TR function, only a whole-genome binding analysis can reveal the extent of TR/TF binding site overlap and functional significance of TR/TF interactions.

To begin to understand links between genomic organization of TR binding sites and TR dependent gene regulation, we characterized genome-wide TR binding events using a BioChIP approach [44]. Our findings point to different requirements for TRβ at T3 induced target genes versus repressed genes or genes that respond predominantly to unliganded TRs. We also observe unexpected features of TR binding site distribution around T3-induced genes, complex TRE composition and obtain evidence for TR cooperation with several heterologous TFs. Better understanding of influences of genomic context upon TR action will help us determine why TR regulates different genes in different ways.

## Materials and Methods

### Reagents

Sources of reagents are as follows: primers from Integrated DNA Technologies (Coralville, IA), TRβ1 antibody from Pierce Biotechnologies (MA1-216, Rockford, IL), acetylated histone H3 K9 antibody from Cell Signaling Technology (#9671, Danvers, MA), M2-Streptavidin beads from Life Technologies (Carlsbad, CA), T3 from Sigma-Aldrich (St. Louis, MO), HepG2 cells from the American Type Culture Collection (Manassas, VA), Fugene HD from Roche Applied Science (Penzberg, Germany).

### Plasmids

Reporter constructs were generated by mutagenesis of pGL4.13 [MinP] (Promega, Madison, WI), using primers containing TREs described in Table S1. The adm proximal promoter reporter was purchased from Switchgear Genomics (Menlo Park, CA). BIRA and BLRP-TEV vectors were provided by David Moore, Baylor College of Medicine, Houston, Texas. To generate BLRP-TEV-TRβ, human TRβ1 cDNA (NM_000461) was PCR amplified with primers bearing sequences complementary to the multiple cloning region of BLRP-TEV vector (5′-aGCCTCGACGGTACCGATATCCTCGAgTGACTCCCAACAGTATGACAGAAAATGGCCTTA-3′) [44], [45]. Products were sequenced and inserted into the BLRP-TEV vector using a modified version of the standard Quickchange mutagenesis protocol (Stratagene, Santa Clara, CA).

### Cell Culture and Transfection

TRβ-BioChIP cells were created by stable transfection of TRβ and BiRA expression vectors and subjected to dual selection. HepG2 cells and derivatives were cultured in DMEM, with Penicillin and Streptomycin and subcultured every 2–3 days. Prior to transfection assays, cells were supplemented with resin-stripped FBS to remove T3 and transfected with constructs indicated and luciferase reporter activity was normalized by cotransfection of pRL which contains the open reading frame of renilla luciferase.

### BioChIP and ChIP-PCR

BioChIP was performed as described [44]. Briefly, cells were plated in DMEM containing resin-stripped FBS, treated with 100 nM T3 and crosslinked with 1% Formaldehyde and chromatin fragmented with a Bioruptor sonicator (Diagenode). Binding and washing steps were performed as previously described [44] and resulting samples analyzed by QPCR with indicated primers (Table S2) or by sequencing using the methods indicated below. Samples were used in ChIP analyses were analyzed in triplicate, and three pooled replicate samples were used for ChIP-Seq analysis.

### Sequencing

Solexa libraries were prepared using the NEBNext DNA Sample Preparation Kit (NEB) and Illumina PE adapters (Illumina) from three pooled replicates of control cell line and TRβ-expressing cells +/−T3. Libraries were sequenced on a Solexa GAIIx following standard procedures. Solexa sequencing data was aligned to HG19 with Bowtie 0.12.7 [46]. Binding peaks were analyzed using QuEST 2.4 [47].

### Sequence Data Analysis

Genome-wide binding data was analyzed with utilities in the Cistrome Portal [48]. Patterns of genome-wide binding were analyzed by [48]. Sequence motif analysis was completed with MEME analysis [49] and custom Scripts (Table S3). Gene ontological analysis was completed with Gene Codis [50].

### RNA Expression Analysis

For arrays, RNA samples, including three replicates for each treatment group, were extracted using the Qiagen RNeasy kit (Valencia, CA). cRNA was prepared with the Totalprep96 kit (Life Technologies, Grand Island NY) and hybridized to Human HT-12 V4.0 gene expression beadchips (Illumina), according to manufacturers' instructions. Bead Intensity values were extracted by Genome Studio, and processed with the Lumi package of Bioconductor [51], by quantile normalization of log2-transformed expression values, with an offset value of 50; fold changes were calculated for each target, including correction for multiple hypotheses. Realtime PCR was used to analyze gene expression levels using the primer sets (Table S4) and standard SYBR Green reagents (Roche) on a Roche 480 Instrument.

### EMSA

Human TRβ and RXRα DBD-LBDs were expressed as described below. Double-stranded oligonucleotides were labeled by standard polynucleotide kinase methodology and incubated with cellular extracts and 1 ug of poly(dI-dC) (Sigma), in a binding buffer containing 25 mM HEPES, 50 mM KCl, 1 mM dithiothreitol, 10M ZnSO4, 0.1% Nonidet P-40, 5% glycerol. After 30 minute incubation at room temperature, complexes were resolved on a 5% non-denaturing gel and visualized by autoradiography. For some experiments, we used infrared Dye EMSA in which TREs possess a 5′ amine moiety on their forward strand and were labeled with 2X-excess of IRDye800-NHS-esther (Li-Cor Biosciences, Lincoln, NE) and purified by silica column. Complexes were resolved on a 5% non-denaturing gel and visualized on a Li-Core Odyssey Imaging System.

### Protein Purification

RXRα or TRβ DBD-LBD was expressed in a BL21 DE3 Escherichia coli strain (Invitrogen), using plasmids bearing these ORFs downstream of a 6X his tag, under control of a T7 promoter, based on the pet28a vector (Merck, Whitehouse Station, NJ). Protein expression was induced overnight at 18 degrees Celsius with 0.2 mM Isopropyl-β-D-thiogalactopyranoside (IPTG). Bacteria were pelleted and lysed by sonication in lysis buffer (20 mM Tris pH 8.0, 0.5 mM NaCl, 100 µM phenylmethylsulfonyl fluoride (PMSF)). Protein was purified with a nickel-IDA agarose (Sigma-Aldrich St. Louis, MO). TRβ was expressed *in vitro* using the T7 Quick-Coupled Transcription Translation System, according to manufacturer's instructions (Promega, Fitchburg, Wisconsin).

### Mice

Indicated mouse liver samples were harvested in T-PER tissue lysis buffer with added protease inhibitor cocktail (Pierce Bioreagents) from 4 week-old C57 mice maintained on standard chow diet, sacrificed after overnight fast. All procedures were conducted with full approval of the TMHRI Institutional Animal Care and Use Committee.

## Results

### BioChIP-TR System

We used BioChIP [44] to capture TR genome-wide binding events. We employed a dual-stable selection protocol to express human TRβ1 bearing an in frame N-terminal BLRP tag and the *E. coli* BirA biotin ligase in HepG2 hepatocyte cells (referred to hereafter as B7B cells). We isolated pools of transfected HepG2 cells and verified TRβ expression by real-time PCR (Fig. S1A). Selected cells displayed approximately eight-fold elevation of TRβ transcripts relative to low levels of TRβ mRNA observed in control HepG2 cells that were stably transfected with BirA and empty vector (shown) and parental HepG2 cells (not shown). TRβ transcript levels were unaffected by T3 and there were no obvious changes in TRα transcripts. Western analysis confirmed that B7B cells expressed increased TRβ protein versus control HepG2 cells (Fig. S1B). However, it was notable that TRβ protein expression levels were lower than seen in equivalent amounts of mouse liver extracts indicating that this expression strategy did not result in supraphysiologic TRβ protein levels.

Exogenously expressed Biotin-tagged TRβ displayed normal DNA binding and transactivation function (Fig. S1C–F). Gel shift analysis revealed increased TRE binding activity in extracts of B7B cells versus control HepG2 cells and that this complex supershifted with antibodies against TRβ and RXRα (Fig. S1C, left panel), with an extent of superhshifting similar to that seen in previous studies [52], [53], co-migrated with *in vitro* translated RXRα-TRβ heterodimer and not complexes formed by *in vitro* translated TRβ or RXRα alone (center panel) and displayed a modest mobility shift in the presence of T3 (right panel). Thus, TRβ adopts standard heterodimeric form in these cells. Transfection of TRE-dependent luciferase reporters (DR-4 and ER-6) into the B7B cells revealed amplification of T3-dependent luciferase activity versus control HepG2 and this effect displayed T3 dose requirements that resemble prior results obtained with stably expressed TRβ in this cell type [54] (Fig. S1D). We also observed enhanced T3 activation of known direct human TR target genes, low density lipoprotein receptor (LDLR) [55], B-cell lymphoma 3 (BCL3 [56]) (Fig. S1E) relative to weak T3 induction seen in HepG2 cells that express BiRA alone (Fig. S1E) and parental HepG2 cells (not shown). For LDLR, this T3-dependent increase was also reflected in increased protein levels as judged by western analysis (Fig. S1F). Thus, overexpressed TRβ displays normal heterodimer formation and transcriptional activity in this cell background.

There was close concordance between patterns of T3 response in B7B cells and HepG2 cells that stably express Flag-tagged TRβ and were previously created by our group [49]. Both the new B7B cells and Flag-TRβ cells expressed similar levels of TRβ transcripts (Fig. S2A) and exhibited very similar levels of T3 induction of endogenous target genes (Fig. S2B and not shown) and a standard DR-4 driven luciferase reporter (Fig. S2C). Thus, properties of B7B cells resemble those of other HepG2 cells that stably express TRβ.

### Enrichment of TRß near transcribed genes

We performed ChIPseq analysis of TRβ DNA binding. Briefly, we treated B7B cells +/−T3, precipitated biotinylated BLRP-TRβ and associated DNA fragments using a streptavidin-based purification system and analyzed TRβ associated DNA sequences (Methods). Data were mapped to the human genome (hg19) using Bowtie 0.12.7 [46], and genomic regions that were over-represented in TR-enriched samples, relative to unenriched control DNA, were assessed using the QuEST package, version 2.4 [49].

**Figure 1:**
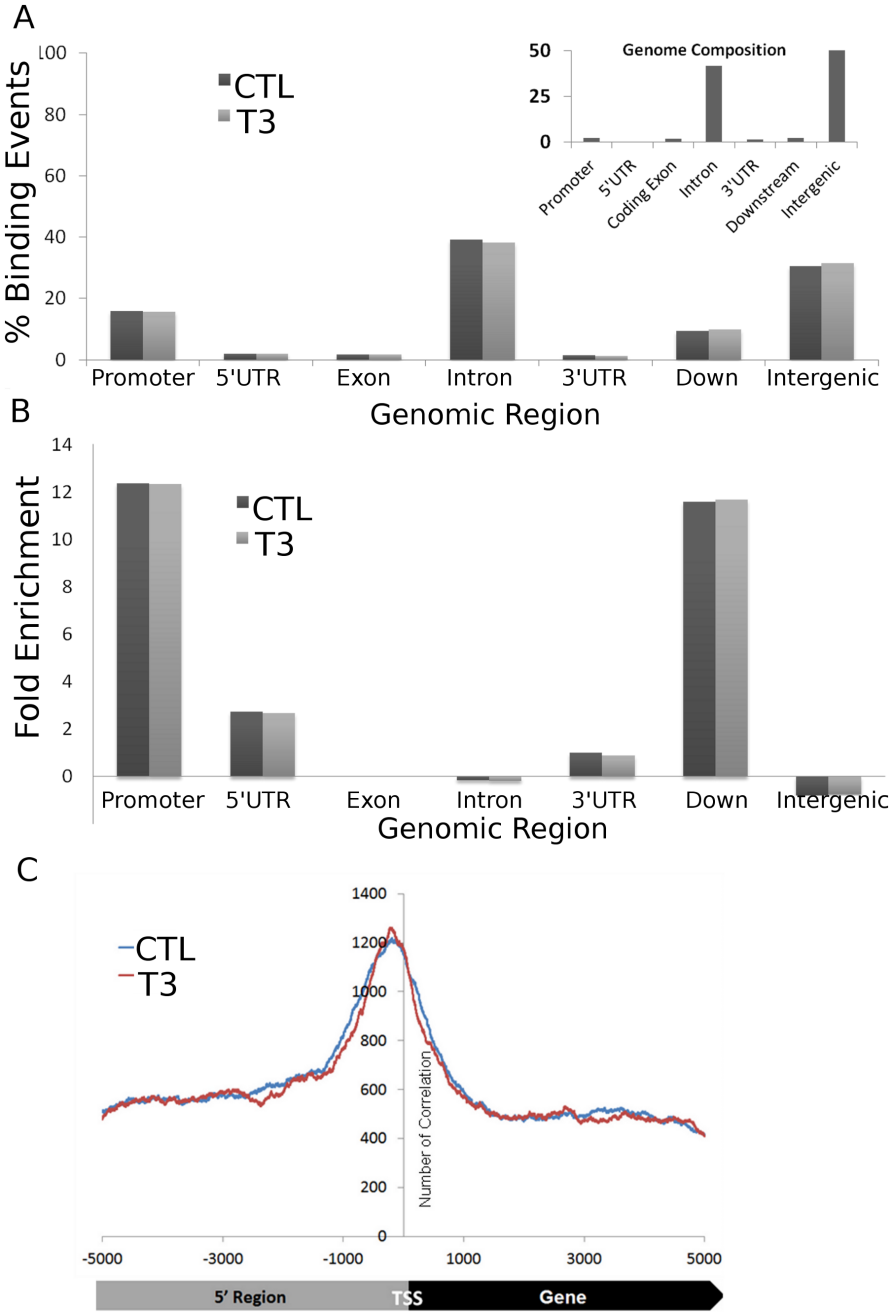
Characterization of genomic binding events. A. Distributions of TRβ binding peaks across specific genomic regions in the absence (black) and presence (grey) of T3. B. Bar graph representing relative enrichment of TRβ-bound regions within genomic intervals specified. Gene-proximal regions, including promoter regions, 5′UTR regions and downstream regions were highly enriched in TRβ-bound regions of the genome. C. Frequency distribution plot of binding events in regions proximal to transcriptional start sites (TSS) +/−T3 (blue and red, respectively). The x-axis represents nucleotides upstream and downstream of the TSS, y-axis represents numbers of binding events.

Genomic distributions of TRs were similar to those of other NRs. We detected 5,791 TRβ binding events in untreated TRβ BioChIP cells and 6,792 in equivalent samples treated with T3 (Fig. 1, S3 and Table S5); within the range of NR binding site representation in other ChIPseq studies. Distributions of TR DNA binding peaks were also similar to other NRs; around 15% of TRβ binding events mapped to proximal promoters (within 5 KB upstream of transcriptional start sites, TSS) and large fractions of TRβ peaks were located within introns or intergenic regions with the remainder associated with downstream region, 5′ untranslated region (UTR), 3′UTR and exons (Fig. 1A). There were no changes in overall distribution of TRβ +/−T3.

Even though TRβ is widely distributed throughout the genome, we observed striking enrichment of TRβ near transcribed genes. We compared numbers of TRβ binding events assigned to different genomic regions (Fig. 1A) versus percentage representation of each region within total genome sequences (Fig. 1B). This revealed a 12-fold enrichment of TRβ binding within proximal promoters, 2-fold enrichment within 5′UTR regions and 11-fold enrichment at immediate downstream regions. Conversely, intergenic binding events were less frequent; around 50% of the genome is classified as intergenic and only around 40% of binding events mapped to these regions (Fig. 1B). There was no enrichment of TRβ binding events within exons or introns relative to their overall genomic representation. The preference of TRβ for proximal promoters of transcribed genes was also reflected in a frequency distribution graph, which revealed significant enrichment of TRβ binding around the TSS (Fig. 1C). Similar to overall binding site distribution, T3 did not affect enrichment of TRβ binding events near actively transcribed genes (Fig. 1B) or the TSS (Fig. 1C). Thus, TRβ binding events are widely distributed throughout the genome but enriched near transcribed genes. Moreover, T3 does not trigger large scale redistributions of TRβ between different functional regions of the genome.

### TR Binding Events are Associated with T3-Induced Genes

**Figure 2:**
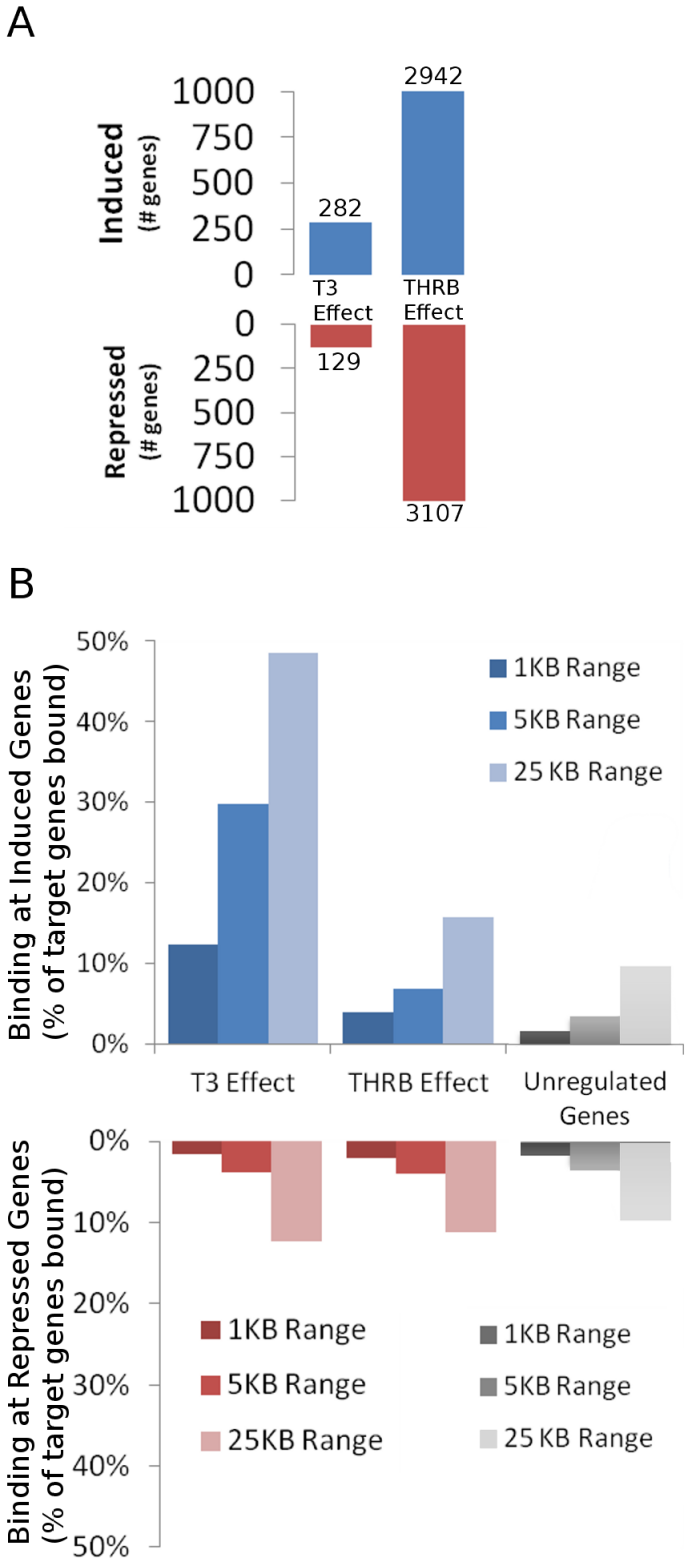
Links between TRβ Binding and regulatory events. A. Bar graph representing numbers of genes that display positive regulation (upper panel) or negative regulation (lower panel) that met statistical significance and an arbitrary +/−1.7-fold cut-off in an array-based analysis of TRβ-BioChIP cells +/−T3 or in TRβ-BioChIP cells versus parental cells that lack TRβ (THRB effect). B. Bar graph representing percentages of TRβ binding events within 1 KB, 5 KB or 25 KB of the TSS of T3 induced, TRβ induced or unaffected genes (upper panel) or T3 or TRβ repressed genes (lower panel). Progressively lighter shading in the bar graph columns represents increasing distance from the TSS.

To understand relationships between TR binding events and target genes, we compared TRβ binding with TRβ/T3 dependent changes in gene expression. We treated B7B cells with T3 for 8 hrs; which allows induction of a large number of T3 induced genes through direct regulatory events in HepG2 cell backgrounds [49]. We performed an array-based analysis of gene expression and detected 411 T3-regulated genes with at least 1.7 fold regulation (adjusted P<0.05), including 282 induced genes and 129 repressed genes. Large numbers of genes also displayed changes in basal activation or repression in the presence of unliganded TRβ versus control cells (TRβ effect; Fig. 2A). Numbers and identities of T3- and TRβ-dependent genes were consistent with previous studies of HepG2 [54], [57].

There was a high prevalence of TR binding events near T3-induced genes. More than 10% of T3 target genes contained a TRβ binding site within 1 KB of the TSS relative to <1% of unregulated genes. The percentage of T3 target genes associated with binding events increased as larger distances were considered; close to 50% of T3-induced genes displayed TRβ binding within 25 KB of the TSS whereas less than 10% of unregulated genes displayed binding events (Fig. 2B). Searches of the human genome for consensus TREs (DR-4, IP-6) using custom in house algorithms (supplementary material) found no evidence for enrichment of any of these elements near T3 induced genes versus their overall representation within the genome (not shown). Thus, TRβ is enriched near T3 induced genes but this does not reflect clustering of classical TREs at these locations.

By contrast, other classes of TR-regulated genes were not clearly associated with TRβ binding events (Fig. 2B). We observed possible weak enrichment of TRβ binding sites near genes that were activated by unliganded TRs (upper panel, TRβ effect); around 15% were associated with nearby TRβ binding sites versus 10% of unregulated genes. More surprisingly, we did not detect obvious TRβ enrichment near genes that were transcriptionally repressed in response to T3 or by unliganded TRs (Fig. 2B, lower panel; Discussion).

### Clusters of TR Binding Events near T3 Induced Genes

**Figure 3:**
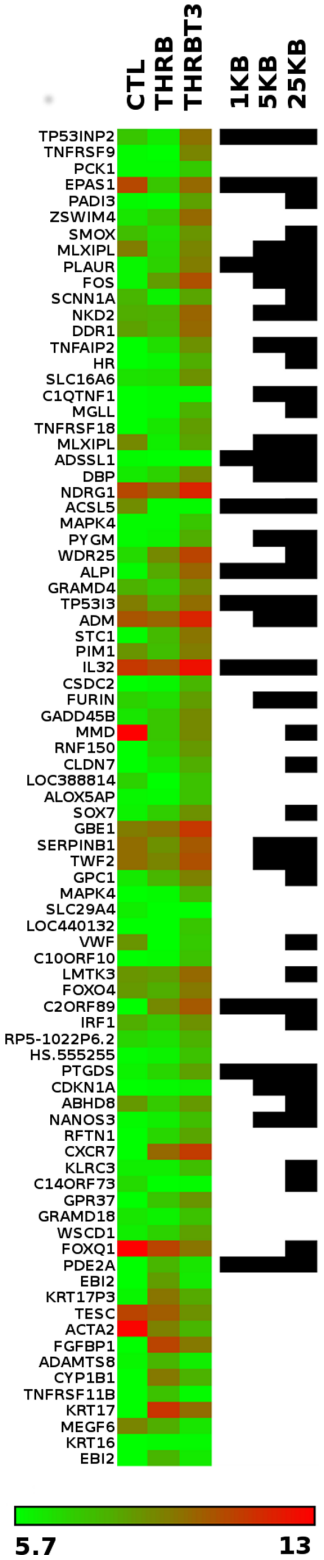
Patterns of TRβ binding and transcriptional regulation. Heatmap depicting log2-transformed expression levels (left) and TRβ binding events within 1 KB, 5 KB or 25 KB of TSS (right) of genes that met statistical significance and an arbitrary +/−2.55-fold cut-off of gene induction in TRβ-BioChIP cells +/−T3. Columns reflect the average of three experimental samples. Expression values in heatmap are as indicated by color scale (bottom, green indicating −5.7-fold repression, red indicating 13-fold induction), and location of binding events within the indicated ranges are depicted by the presence or absence of black bars in the three right-most columns.

We performed a detailed survey of TRβ binding near genes regulated more than 2.5-fold by T3 in the microarray (Fig. 3 and supplementary data). As expected, large numbers of TRβ binding events occurred near highly induced targets; >50% of these genes exhibit TRβ binding within 25 KB and >10% within 1 KB. Genes in this TRβ bound, T3 induced, set were physiologically relevant and representative of TRß's known functions in liver (not shown). There was high correlation between identities of highly T3 responsive genes in B7B cells and observed T3 responses that were insensitive to cycloheximide (CHX) treatment in related HepG2 cells that express flag tagged TRβ (see data in reference [49] and not shown). Further, these genes displayed rapid onset of T3 response in Flag-TRβ cells at early time points (see [54]). This implies that this gene set is highly enriched for direct TR targets. By contrast, only one T3 repressed gene (PDE2A) displayed nearby binding events, within 1 Kb of the transcriptional start site, and no other repressed genes displayed detectable TRβ binding events.

**Figure 4:**
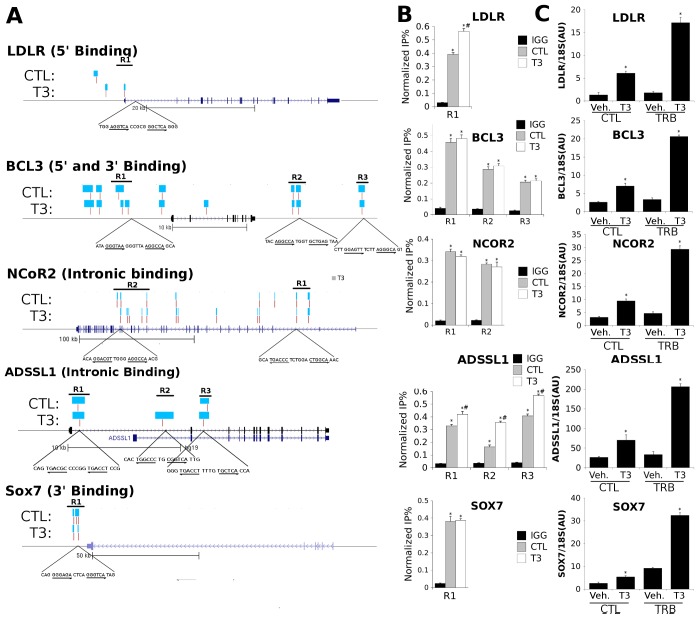
Characterization of TRβ binding near induced genes. A. Patterns of TRβ binding depicted at representations of individual target gene loci (LDLR, BCL3, NCOR2,ADSSL1 and SOX7). Blue bars represent genomic binding regions, and the vertical red lines represent peaks, as classified by QuEST. The horizontal black bars are regions analyzed by ChIP-PCR (locations of primer amplification). Observed binding patterns included 5′, 3′ and intronic binding events, as shown in genomic data tracks (UCSC Genome Browser). Putative regulatory elements, identified through sequence analysis of the genomic regions indicated, are depicted below bound regions in which they occur. B. QPCR of ChIP analysis confirming DNA binding in regulatory regions of genes. C. Realtime PCR analysis depicting enhancement of transcription of individual loci in Fig. 4A by T3 in the presence of TRβ. (*P<0.05 by Student's T-Test).

Closer analysis of patterns of TRβ binding near selected genes revealed clusters of TRβ binding sites in diverse distributions. In Figure 4, TRβ binding events are represented by blue bars defining the extent of sequences precipitated by the BioChIP experiment and red bars representing TRβ peak positions defined by QuEST. We observed TRβ binding near both verified human TRβ target genes LDLR and BCL3 (Fig. S1C) and this mapped to the 5′ region of the transcription unit for LDL-R and to the 5′ and 3′ regions for BCL3 (Fig. 4A). Similar “5′ only” and 5′+3′ distributions were also seen for other genes (not shown and see Fig. S4). Interestingly, for both genes, TRβ peaks detected by BioChIP were close to known TREs in the proximal promoter region [55], [56]). We also observed other types of TRβ binding site distributions near highly induced genes; these included intronic (NCOR2 and ADSSL1) and 3′ only (SOX7). We confirmed TRβ binding at predicted sites by conventional ChIP with an antibody specific for human TRβ (Fig. 4B, amplified regions are represented by black bars in Fig. 4A) and verified that there was increased T3-dependent gene induction versus control HepG2 cells with qPCR (Fig. 4C). Thus, TRβ binds at multiple locations in varied patterns near strongly T3 induced genes and this correlates with enhancement of T3 response.

### Changes in TRβ binding events after hormone treatment

While there were no major changes in overall TRβ distribution +/−T3 (Fig. 1), we detected more TRβ peaks with T3 versus untreated cells (6792 versus 5791; 17% increase). We compared positions of TRβ binding peaks +/−T3 in more detail. Surprisingly, the majority of TRβ peaks exhibited hormone-dependency with only 30% completely unchanged (Fig. S3A). We did not observe any specific association of T3-dependent peaks and particular functional regions of the genome (Fig. S3B, S3C); distributions of ligand dependent binding events reflect overall distributions of TRβ (see Fig. 1).

To better understand this effect, we inspected hormone-dependent TRβ binding events near target genes. This approach revealed only modest changes in TRβ peak distribution around transcription units rather than large scale alterations in TRβ binding pattern (see examples in Fig. 4, Fig. S4A). In some instances (see LDLR, Fig. 4A and not shown), there were modest shifts in TRβ peak and footprint position. Further, some called peaks appeared completely hormone-dependent (see ADSSL1 R2, Fig. 4, PDE2A R1, Fig. S4A and not shown). Such changes were not seen at all genes (EPAS1; ACSL5, Fig. S4A). We were able to verify 2-fold hormone-dependent changes in TRβ binding at some locations at which TR binding was completely hormone-dependent by conventional ChIP (Fig. 4B, LDLR; Fig. S4B, PDE2A and not shown). Conversely, we found that peaks which appeared unchanged after T3 treatment by BioChIP were generally unaffected by hormone when assessed by this method (Fig. S4B). We therefore suggest that apparent hormone-dependent changes in TRβ binding site distribution (Fig. S3) reflect, at least in part, hormone-dependent changes in positions and size of TRβ binding peaks (Discussion).

### TRβ Binding Peaks Contain Complex TREs

**Figure 5:**
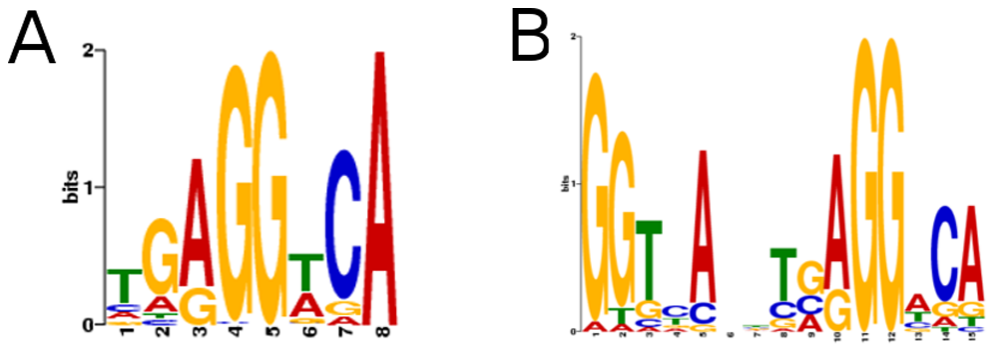
Definition of TRE Consensus. A. Consensus sequence with similarity to the classic TRβ binding half-site discovered by analysis of top 150 peaks in BioChIP analysis. B. The previously defined TRβ consensus obtained from analysis of more than 30 published target gene regulatory elements is shown for comparison at right.

Investigation of sequence composition of genomic regions bound by TRβ revealed elements that resembled the typical TRE consensus within the peaks. A query of the top 150 bound peaks revealed high prevalence of a single TRE half sites (TGAGGTCA) (Fig. 5A), distinct from a previous consensus derived from analysis of a smaller number of TREs which contained two detectable half sites spaced by four bases (Fig. 5B, 28). Interestingly, this previous study also suggested that the paired G residues (TGAGGTCA) were the most important determinants of TRβ binding [30]. While our analysis confirmed that these nucleotides are indeed highly represented, it also revealed higher representation of other nucleotides within the half site than previously defined (TGAGGTCA ).

A more directed query, based on the above results and including all sequences within TR-bound regions, confirmed the presence of canonical half sites in 50% of bound regions (Table 1). A similar query with a degenerate half-site sequence (AGGnCA) produced positive results for more than 90% of bound regions. Although half-sites are common in the genome, there was approximately 2-fold enrichment of both consensus and degenerate half-sites in TRβ peaks relative to their overall representation.

**Table 1:** Occurrence of binding motifs in TRβ-bound peaks.

	Motif	Untreated	Enrichment	T3-Treated	Enrichment
Halfsite	AGGTCA	50.32%	2.10	48.04%	2.08
Halfsite	AGGnCA	91.71%	8.68	90.75%	8.51
DR4	AGGnCA-4-AGGnCA	1.91%	17.57	1.69%	14.45
DR4	AGGnnn-4-AGGnCA	11.36%	26.00	11.09%	25.15
ER6	nnnCCT-6-AGGnCA	8.87%	4.43	8.01%	4.26
IP0	nnnCCT-0-AGGnCA	3.45%	1.68	3.28%	1.65
IP1	nnnCCT-1-AGGnCA	11.81%	6.10	10.92%	5.38
Neg1	TTTGGG	43.57%	1.76	42.37%	1.75
Neg2	CCCCTCAGGCGC	0.02%	2.09	0.01%	1.87

Close investigation of TRE sequences within TRβ peaks revealed that half-sites were usually associated with a second, less well conserved, half-site and that organization of these elements commonly resembled half-site configurations of classical TREs, including DR-4, ER-6, IP-0 and IP-1 (Table 1). Genome wide, there was 14-fold enrichment of putative DR-4 elements in TRβ binding peaks and 4–5 fold enrichment of ER and IR elements. We also detected high representation of canonical DR elements with unusual spacing (DR-0 to DR-12) and variations in half-site spacing of ERs and IRs, although degeneracy in the 5′ half site can mean multiple interpretations of organization of these TREs (not shown).

**Figure 6:**
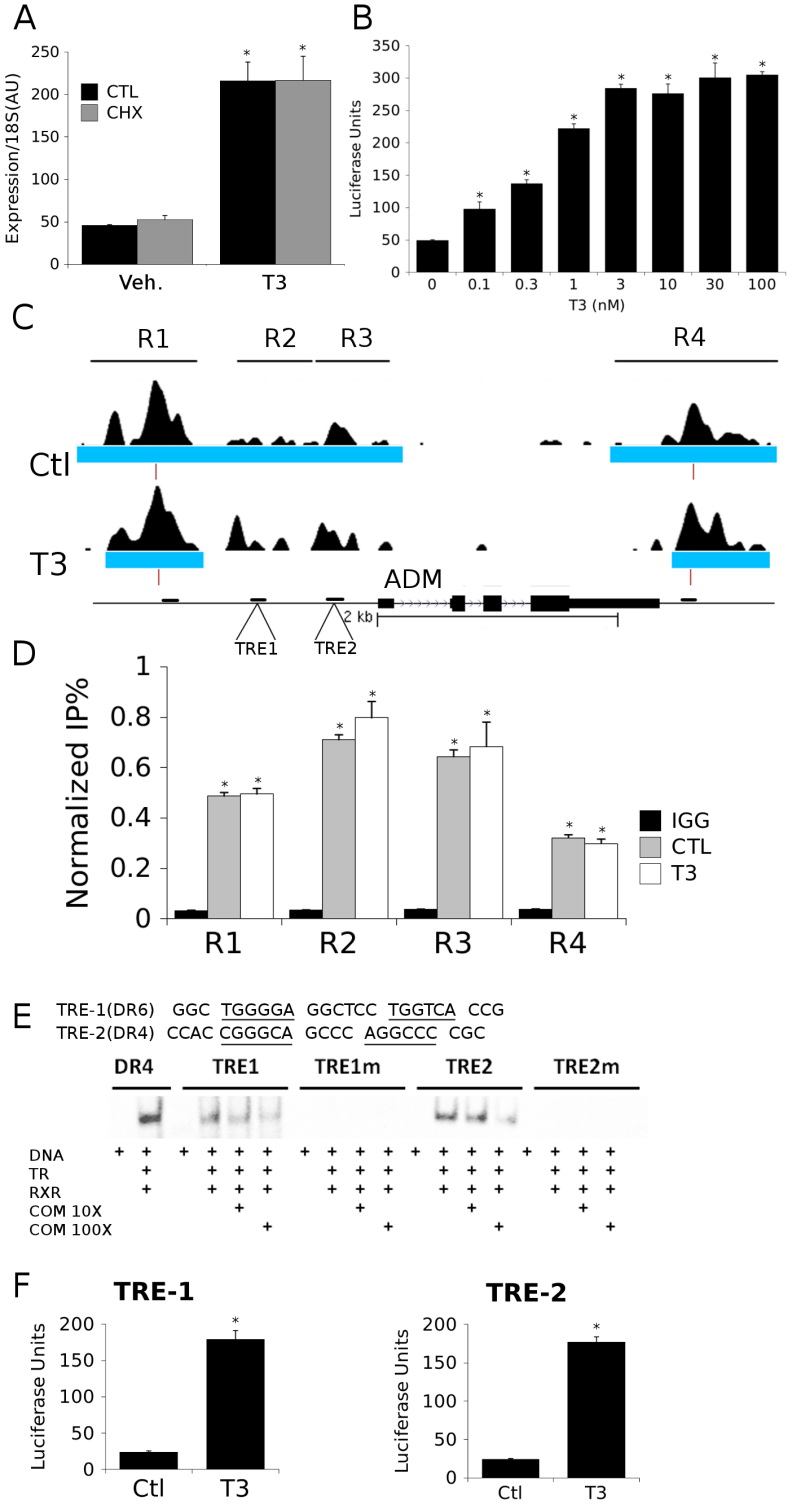
Links between TR Binding and Adm Transcription. A. Graph showing results of realtime PCR analysis of adm transcription in the B7B cells after six hours of T3 treatment +/−10 µg/ml CHX cotreatment of B7B cells. B. Patterns of TRβ binding peaks at the *adm* locus (UCSC Genome Browser), in similar format to Fig. 4. TRβ binding events clustered into four regions (R1, R2, R3, R4), upstream and downstream of this transcript, as well as a substantial amount of binding immediately proximal to the transcriptional start site. C. Binding of TRβ was confirmed by realtime ChIP PCR analysis in B7B cells at the regions indicated (ChIP primers are depicted by horizontal bars in B). D. The proximal promoter region of *adm* (corresponding to R2) conferred T3-dependent increases in luciferase activity upon a standard reporter after transfection into B7B. E. Results of gel shift confirming direct TRβ binding to two putative response elements, designated TRE-1 and TRE-2 that were found in R2 at positions marked in Fig. 6B. Individual lanes show shifts obtained with elements and RXRα-TRβ +/− competitor DNA or mutated versions of both elements. F. Luciferase reporter assays confirming that TRE-1 and TRE-2 confer T3 responsiveness on a reporter gene. (*P<0.05 by Student's T-Test).

**Figure 7:**
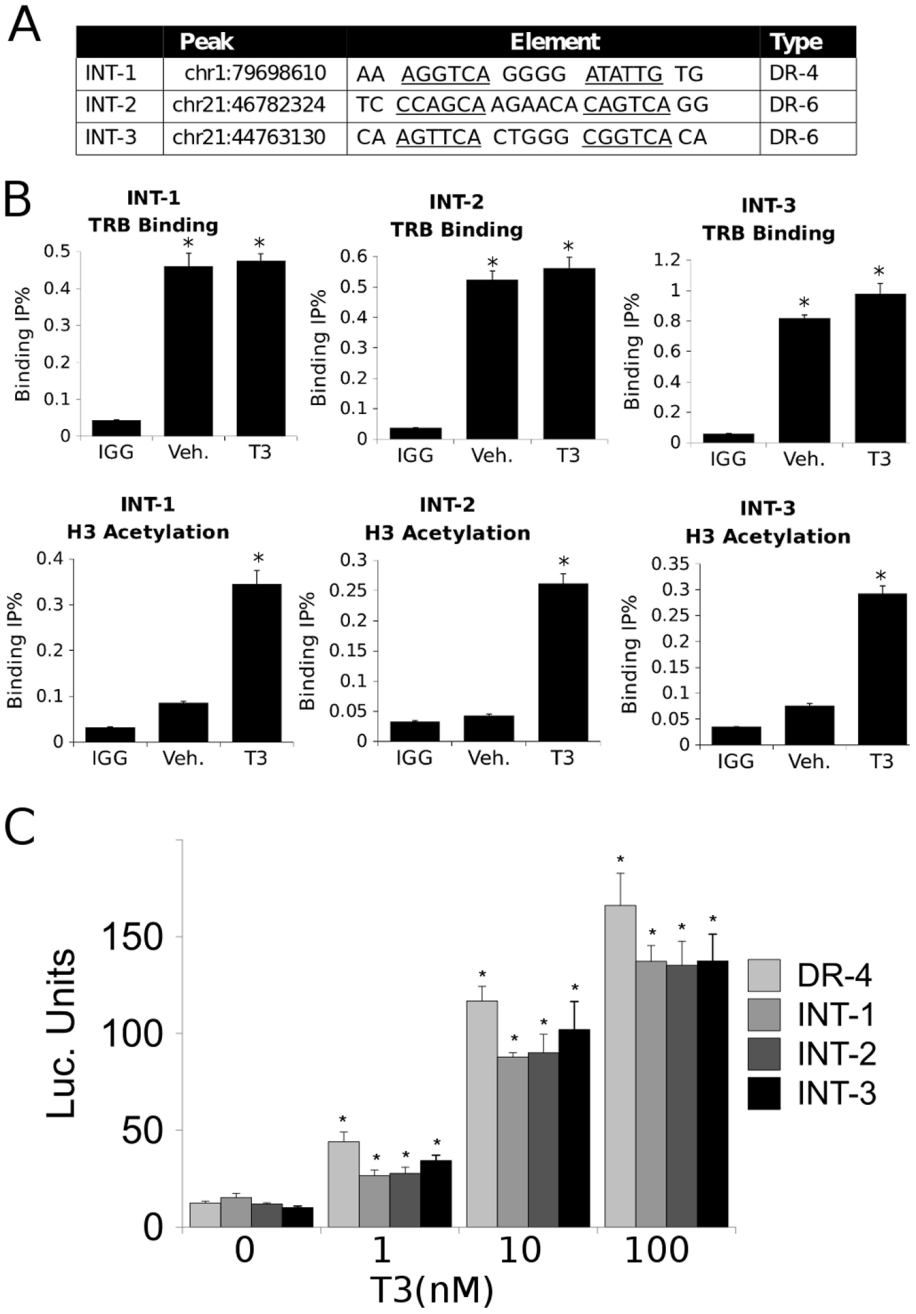
Intergenic binding events. A. Three intergenic binding peaks were selected and analyzed for the presence of recognizable TR binding motifs (sequences of motifs listed). B. Results of qPCR ChIP analysis confirming binding of TRβ to the intergenic regions depicted in Fig. 7A (top panels) and induction of H3 acetylation near sites (bottom panels). C. Results of luciferase reporter assays, with indicated constructs containing intergenic elements described in Fig. 7A, confirming that each element confers T3 induction. (*P<0.05 by Student's T-Test).

To understand whether putative elements identified by computational approaches were functional TREs, we performed further analysis of TRβ binding near the adrenomedullin (adm) gene, which is a verified TR target in rodents [58], [59]. It is also known that adm levels increase in hyperthyroid human patients [60], [61], but the molecular basis of the latter effect have not been previously characterized. We confirmed that T3-dependent transcriptional activation of human adm was unabated by the translation inhibitor CHX in TRβ-BioChIP cells, proving that it is a direct target (Fig. 6A) and that an adm promoter (−1 KB) driven luciferase reporter also displayed T3 induction after transfection into B7B cells (Fig. 6B). There were TRβ binding footprints (blue bars) and called peaks (red bars) in the proximal promoter and immediately downstream of the gene (Fig. 6C) and we verified these binding events with conventional ChIP using indicated primers (R1–R4; Fig. 6D). Use of a custom position-weighted matrix-based comparison with previously characterized TR regulatory sites revealed two probable TREs within R2, a DR-4 element and a DR-6 element that lay within 1 KB of the TSS (Fig. 6C). Binding of both elements to TRβ was confirmed by EMSA (Fig. 6E) and both robustly activated transcription from a luciferase reporter (Fig. 6F). Thus, T3 induction of human adm involves TRβ binding to TREs in the proximal promoter, along with other sites. We were routinely able to identify TREs in other TRβ peaks and verify function (see Fig. 7, Figs. 4 and S1 and manuscripts in preparation). We conclude that TRβ is commonly recruited to DNA through interactions with TREs that contain canonical or variant AGGTCA half sites, albeit with diversity in spacing and orientation.

Finally, we considered representation of putative negative response elements, including “TTTGGG” and “CCCCTCAGGCGC” [35], [36], [39] in TRβ binding sites (Table 1). Both elements were enriched in TRβ peaks relative to their overall genomic representation (1.7 and 1.9 fold respectively). Investigation of TRβ peaks that were proximal to a highly negatively regulated gene (PDE2A see Fig. 3), however, failed to reveal possible nTREs, although we did observe classical TRE-like sequence elements that resembled DR-4 sites (Table S1). Further, consideration of peaks detected near other negatively regulated genes (SOX9 etc.) that not meet cutoffs indicated in Figure 3 failed to reveal any nTRE-like sequences in most cases, the sole exception being C9ORF169 (Table S6). Thus, hormone-dependent suppression of TR target genes in HepG2 cells is not commonly associated with detectable TRβ binding to nearby nTREs (Discussion).

### Intergenic TRβ Binding Sites are functional TREs

Since more than 30% of TRβ binding events occurred in intergenic regions (Fig. 1A), similar to other NRs, we tested the ability of some of these sites to regulate transcription. We verified TRβ binding to three intergenic peaks with ChIP (Fig. 7A, 7B). We also used ChIP to confirm that T3 induced an activating histone acetylation near each peak (H3Ac, Fig. 7B). We identified putative TREs within each peak (Fig. 7A) and confirmed activity by EMSA (not shown) and assays of luciferase activity (Fig. 7C). Thus, intergenic binding events can induce an active chromatin state in response to T3 and co-localize with functional TREs.

### Colocalization of TR and TF Binding Sites

Finally, we determined whether TRβ might associate with heterologous TFs in composite modules. We examined TRβ peaks for over-representation of TF binding sites, using a comprehensive query of regions with known TF consensus sequences in the JASPAR database [54], with Seqpos (http://cistrome.org) and these are listed in Table 2, with the most commonly associated factors listed at top.

**Table 2:** Occurrence of previously identified binding motifs in TRβ-bound peaks.

ID	Name	Family	Z-Score
MA0099	JUN	BZIP	−32.85
MA0160	NURR1	NHR	−27.44
MA0018	CREB1	BZIP	−26.47
MA0071	RORA	NHR	−27.6
MA0159	RARA	NHR	−15.27
MA0112	ESRA	NHR	−15.21
MA0150	NFE2L2	BZIP	−13.43
MA0258	ESRB	NHR	−13.3
MA0115	LXRB	NHR	−12.17
MA0043	HLF	BZIP	−10.38
MA0003	TFAP2A	AP2	−10.37
MA0074	VDR	NHR	−9.72
MA0017	COUPTF1	NHR	−8.62
MA00139	CTCF	GO	−7.91
MA0066	PPARG	NHR	−7.76

We identified a variety of NR binding sites, including RAR, VDR, PPAR, RORα, ERα, ERβ and Nurr1, all of which consist of canonical AGGTCA half-sites of distinct spacing (e.g., RAR binds to DR-5 elements, VDR binds DR-3 and PPARs bind DR-1). While we cannot exclude the possibility that TRs associate with these NRs, the simplest interpretation of this finding is that these elements are atypical TREs, consistent with promiscuity of TRβ DNA binding, see Discussion.

We also observed co-localization of TRβ binding sites with consensus binding sites for several heterologous TFs. These include AP-1 (jun), CREB, p53 and CTCF, all of which have been previously shown to associate with TRβ (Table 2). Additionally, we detected association of TRβ with NFE2L2 binding sites (also known as NRF2), involved in regulation of the antioxidant response pathway, AP2 and others. There were no major changes in preference of TRE/TF binding site co-localization near TRβ peaks +/−T3 (Fig. S2). Thus, our findings support previous studies which suggest that TR cooperates with a small subset of heterologous TFs that includes AP-1 and CTCF and also identify new candidates for consideration.

## Discussion

In this study, we have assessed genomic distributions of TRβ in a human liver cell line to understand how TRβ binding patterns are related to T3 and TR-dependent changes in gene expression. We used HepG2 cells that express exogenous BLRP-tagged TRβ and enzymatic machinery required for biotinylation (BirA) to examine TRβ binding [44] because we and others have found that HepG2 cells display highly reproducible and widespread T3-responses at physiologically relevant genes [54], [62]. Stable TR expression is necessary because TR mRNA levels are greatly reduced in liver cell lines and primary cultures versus native [57]. Given this requirement, we selected the BioChiP system because it allows straightforward, high stringency purification of TRβ [44]. Interestingly, many of our findings resemble those obtained with exogenous TRs in a mouse neural cell line suggesting that combined results of these approaches are revealing at least some fundamental principles of TR genomic distributions and actions [7], [19].

Our findings indicate that mechanisms of T3-dependent gene induction often conform to prevailing models of TRβ action. While TRβ distribution resembles that of other NRs, with TRβ peaks spread widely through the genome and often far from obvious TR target genes, we nevertheless detect striking enrichment of TRβ at proximal promoters, the TSS and immediate downstream regions of transcribed genes (Figs. 1B, C) and more than 45% of T3 inducible genes are associated with TRβ peaks (Fig. 2B). Further, >90% of TRβ peaks contain a TRE half-site, there is significant enrichment of DR-4 elements and other known TRE configurations and we commonly identify putative TREs within bound regions (see Figs. 4, 6 and 7 and Table S1,S6) and are able verify TRE function (Figs. 5 and 7 and manuscripts in preparation).

More surprisingly, we failed to detect obvious TRβ enrichment near T3 repressed genes. Further, while we do observe enrichment of putative nTREs in TRβ peaks, we have been unable to link specific nTREs to nearby highly repressed targets. Both findings appear surprising in light of previous results which show that TRs are directly recruited to negatively regulated genes such as [34]–[36], but we think that our results do not contradict these findings and instead point to the existence of alternate mechanisms of TR gene repression that are active in HepG2 cells. We previously found that large proportions of negatively regulated genes are inhibited by CHX in HepG2 cells, implying that new protein synthesis is often required for transcriptional repression in this context, and we therefore suspect that many individual instances of T3 repression in HepG2 cells could involve secondary responses. Other phenomena may also be at play, including TR/T3-dependent remodeling of coregulator complexes that interact with multiple genes. Clearly, this issue will require further investigation and it is not clear whether a similar preponderance of indirect effects will also be a feature of T3-dependent negative regulation *in vivo* or whether this is restricted to the HepG2 system. We also failed to detect significant TRβ enrichment near genes that respond to unliganded TRs. We think that this could also reflect a preponderance of indirect response to active unliganded TRs.

While TR actions at many T3 induced genes often appear to be mediated by proximal binding events, our findings emphasize that mechanisms of T3 induction are not always straightforward; around 50% of T3 induced genes lack proximal TRβ binding peaks, raising the possibility that such instances are explained by either long-range regulatory events or local binding events which are too ephemeral for detection through the currently employed method. While we expect that the latter possibility will be true in some cases, we surveyed several proposed TRβ binding sites that were not detected in our ChIPseq studies, including within the pck1 promoter, and have concluded that functional TREs are not present and that we did not overlook TRβ binding in these cases (not shown). Another possibility, mentioned for T3 repression, is that some T3 induced genes are indirect TR targets that respond to primary TR-dependent changes in levels of other TFs. We suspect that this only applies to a small number of examples of T3 induced genes that lack TRβ binding sites; genome wide assessment of CHX sensitivity in HepG2 indicates that most T3 induced genes are direct TR targets [54]. We therefore instead favor the idea that this subset of T3 induced genes is subject to extremely far distant regulation by functional intergenic TRβ binding sites [4], [17]. While it is intriguing to suggest that putative intergenic TREs, similar to those in Fig. 7, are responsible for these long range effects, this idea must be treated with caution. The fact that these elements are not near known genes but activate luciferase from a proximal location suggests that they have the capacity to be functional but their true role in the context of the whole genome is unclear. While the increase in acetylation seen nearby these locations with T3 is interesting, it is also conceivable that TRβ binds adventitiously to elements that happen to lie within “open chromatin” rather than to true functional elements in this system.

The ChIPseq approach confirms and extends previous suggestions that TRE organization is variable [63], [64]. We have been able to define a consensus TRE half-site that is commonly represented with TRβ peaks, found that consensus half-sites (AGGnCA) occur in >90% of TRβ peaks and observed enrichment of classical DR-4 elements and, to a lesser extent, other canonical TREs. However, total representation of DR-4, IR-0 and ER-6 elements is much lower than total TRE half-site representation (Table 1). This discrepancy is a consequence of high representation of TRE-like sequences with non-canonical half-site spacing within TRβ peaks (Table 2) and probably also explains why we detected large numbers of binding sites for other NRs that interact with AGGTCA half-sites. This raises obvious questions about: i) the structural basis of flexibility of TR/TRE recognition, ii) connections between promiscuous DNA recognition and gene-specific variations in TR activity and iii) possibilities for NR/TR cross-talk in gene regulation. Our study does reveal differences with the canonical TRE site identified by Chatonnet and coworkers, who observed a DR-4 like consensus in neural cells rather than the half-site seen here [19]. We do not understand whether this difference is related to technical aspects of our study versus that of Chatennet, including TR expression levels, or whether there are true differences in response element recognition patterns in different cell types. This issue will also require further investigation.

Our findings also reveal unexpected features of TR binding site architecture. T3-induced genes are often associated with clusters of TRβ peaks rather than single elements, and there is enrichment of TRβ binding both 5′ and 3′ of transcribed genes and binding sites can be seen within untranscribed regions and within introns. It is not clear whether TRs play distinct roles in gene expression when bound to different locations with respect to the transcription unit; this issue will require further investigation. It is also intriguing that computerized analysis of consensus TREs throughout the genome indicates that they are not obviously over-represented near TR target genes, implying that actual TRβ binding events are dependent on other factors, possibly local states of chromatin modification or binding events of partner TFs. It will be important to understand mechanisms that underlie TRβ binding site selection within the milieu of living cells.

A large proportion of TRβ peaks exhibit some apparent T3-dependency, but we do not believe that this reflects large scale redistribution of TRs in response to hormone. Overall genomic localization of TRs is similar in the absence and presence of T3 (Fig. S3) and reanalysis of data with altered stringency of peak calling did not change our conclusion that overall TR distribution does not change after hormone treatment (not shown). Close investigation of TRβ peaks near target genes revealed relatively modest alterations in TRβ peak position and footprint size rather than large scale appearance or disappearance of TRs from the vicinity of target genes (Fig. 4 and Fig. S4). We found some examples in which apparent changes in footprint size could not be verified with standard ChIP (see adm footprints in Fig. 5). More commonly, however, we were able to verify at least some degree of hormone-dependency of TRβ binding at selected peaks with conventional ChIP. Thus, we favor the idea that there are modest redistributions in TRβ binding after hormone treatment and think that this effect accounts for the large apparent change in TRβ peak distribution after hormone treatment.

Functional significance of verifiable hormone-dependent changes, if any, remains unclear. T3 may promote relocalization of TRβ from inactive DNA pools near a negatively regulated target gene to nearby functional regulatory elements [65]. Our observations suggest, however, that T3-dependent changes in TRβ footprint and peak position are complex and gene-specific with no obvious pattern. We recently showed that hormone-dependent TRβ binding to a TRE within the glucose-6-phosphatase promoter requires TRβ interactions with a gene specific cofactor, the NAD+-dependent deacetylase Sirtuin 1 (SIRT1 [66]). Clearly, more investigation will be needed to understand the potential importance of this unexpected phenomenon in the context of T3-dependent TRβ binding.

Finally, our results allow us to define possibilities for TR cross-talk with other TFs. Fewer than 10% of TRβ peaks lack identifiable TREs, suggesting that DNA independent TRβ recruitment that exclusively involves contacts with heterologous TFs such as AP-1 is relatively rare. However, we do detect strong enrichment of binding sites for TFs that are known to interact with TRs, including AP-1 [37], [38], CTCF [39] and p53 [40], and for other TFs that have not previously been proposed to cooperate with TRs, including NRF2 and AP2. It will be interesting to see whether TR interacts with these factors in composite modules and examine roles of complex elements in T3 response and cross-talk of TRs with other signaling pathways.

In summary, our studies indicate that T3 gene regulation commonly involves proximal TRβ binding events near target genes, but also reveals striking variability in TRE position, distance from the target gene, TRE half-site organization, sequence and spacing and interactions with heterologous TFs. We and others have previously described large variations in precise mechanisms of TR action at different target genes [54], [57], [67]. It is interesting to speculate that these differential effects are linked to variations in genomic context of TRβ binding sites described here.

## Supporting Information

Figure S1 **BioChIP-modified TRβ constructs exhibit similar activity to endogenous TRβ.** A. BLRP-tagged TRβ was expressed in HepG2 cells at a level 8–10 fold higher than that endogenously expressed in parental HepG2 cells, as assessed by realtime PCR, normalized to 18S RNA. B. TRβ protein levels in B7B cells (lanes 5–8, 20 µg cellular protein per lane) were found to be substantially less than that expressed in mouse liver (lane 9, 20 µg protein per lane) as assessed by western blot. C. Modes of DNA binding were assessed by EMSA assays, as indicated. B7B cells elicited mobility shift of a DR4 element (left panel, lanes 6–9), similar to that of previously characterized [54] Flag-tagged TRβ cell lysates (“HG2 FlagB”, lanes 10–13). Supershift of complexes with RXRα or TRβ antibodies indicated the predominantly heterodimeric composition of complexes (left panel, upper band). Analysis of B7B cell lysates, in comparison to in vitro-expressed RXRα and TRβ protein alone (middle panel, lanes 2&3) or combined lysates (lane 4) confirmed a predominantly heterodimeric mode of binding to DR4 elements (lanes 5–7), similar to Flag TRβ lysates (lanes 8–10). Analysis of T3-treated cell lysates revealed a characteristic change in mobility, as compared with untreated lysates, similar in B7B (right panel, lanes 5–7) and HG2 FlagB lysates (lanes 8–10). D. Transcriptional activation of DR4 and ER6 elements were quantified in comparison to controls in luciferase reporter assays in HepG2 cells (left panel) or TRβ-expressing HepG2 cells (right panel). E. Expression of defined TRβ target genes was quantified by realtime PCR of target genes in indicated cell models, normalized to 18S RNA. F. Protein expression of the TRβ-regulated gene LDLR was assessed by western blot in TRβ-expressing HepG2 cells, showing substantially increased protein levels after T3 activation of TRβ.
(TIF)

Figure S2 **Comparison of B7B cells and HepG2-TRβ (Flag) cells.** A. Expression of BLRP-tagged TRβ or Flag-tagged TRβ was 8–10 fold higher than endogenous TRβ expression in HepG2 parental cells and was not affected by treatment with 100 nM T3 for 8 hours, as assessed by realtime PCR, normalized to 18S RNA.Expression of TRα was similar in all samples. B. Expression of defined TRβ target genes was induced with 8 hour T3 treatment to a similar extent in BLRP-tagged TRβ and Flag-tagged TRβ cells, as assessed by realtime PCR of indicated targets, normalized to 18S RNA. C. A DR4 luciferase reporter construct showed a similar transcriptional activation profile after treatment of BLRP-tagged TRβ and Flag-tagged TRβ cells; little activation was observed in HepG2 parental cells.
(TIF)

Figure S3 **Summary of binding events in Trβ-expressing cells.** A. TRβ binding peaks were mapped to genomic regions, using the methods described. Approximately 30% of binding to sites occurred only in the presence or absence of T3, as depicted in the Venn diagram shown. B. Percentages of total binding events within specific regions, as assessed with the CEAS analysis program [68], [69], are shown, including those occurring exclusively in untreated samples, or exclusively in T3-treated samples are indicated. C. Percentages of binding events, which were exclusively observed in untreated or T3-treated samples, was mapped to the genomic regions indicated, using the CEAS analysis program, and depicted in the charts as labeled.
(TIF)

Figure S4 **Characterization of TRβ binding near induced genes.** A. Patterns of TRβ binding were depicted at individual target gene loci, represented using the same format as Fig. 4. Observed binding patterns included 5′, 3′ and intronic binding events, as shown in genomic data tracks (UCSC Genome Browser). B. Patterns of TRβ binding at the regions indicated with ChIP, analyzed by QPCR. (*P<0.05 by Student's T-Test).
(TIF)

Table S1 **Response elements characterized in reported data.** Hormone response elements are listed by their associated genes (Column 1). The DNA sequence of each element is provided, with spaces to indicate half-site position (Column 2), and the assays used to evaluate the elements described (Column 3).
(PDF)

Table S2 **Primers used for chromatin immunoprecipitation.** TRß binding at indicated peaks was assessed by realtime PCR of chromatin IP-enriched samples with indicated primer sets. Primer sets were designed to specifically amplify indicated genomic regions.
(DOC)

Table S3 **Perl script used for analysis of bound sequences.** Sequence composition of TRß-binding genomic regions was analyzed with the included Perl script, confirmed to run on Ubuntu Standard Distribution 11.1, in addition to the analysis procedures described in text. This script analyzes two BED-formatted input files by evaluating the length-normalized bidirectional site occurrences.
(PDF)

Table S4 **Primers used for realtime PCR-based analysis of gene expression.** Gene expression was assessed by realtime PCR with primer sets, designed to specifically assess expression of indicated genes.
(PDF)

Table S5 **Peaks.** Peaks of TRβ binding are listed for each sample by start and end position. Genes proximal to peaks are listed by name, strand and distance from peak. Peaks over-represented in TR-enriched samples, relative to unenriched control DNA were assessed using the QuEST package, version 2.4.
(DOC)

Table S6 **Negatively regulated genes and associated regulatory elements.** Regulatory elements proximal to genes with negative regulation after T3 treatment (Column 1 and 2) are listed according to their genomic position (Column 3), type of element (Column 4) and sequence (Columnn 5).
(XLS)
