# Supplementary figures and images for: Genome-Wide Binding Patterns of Thyroid Hormone Receptor Beta
Source: PLoS One. 2014 Feb 18;9(2):e81186. doi: 10.1371/journal.pone.0081186 (PMC3928038; doi:10.1371/journal.pone.0081186)

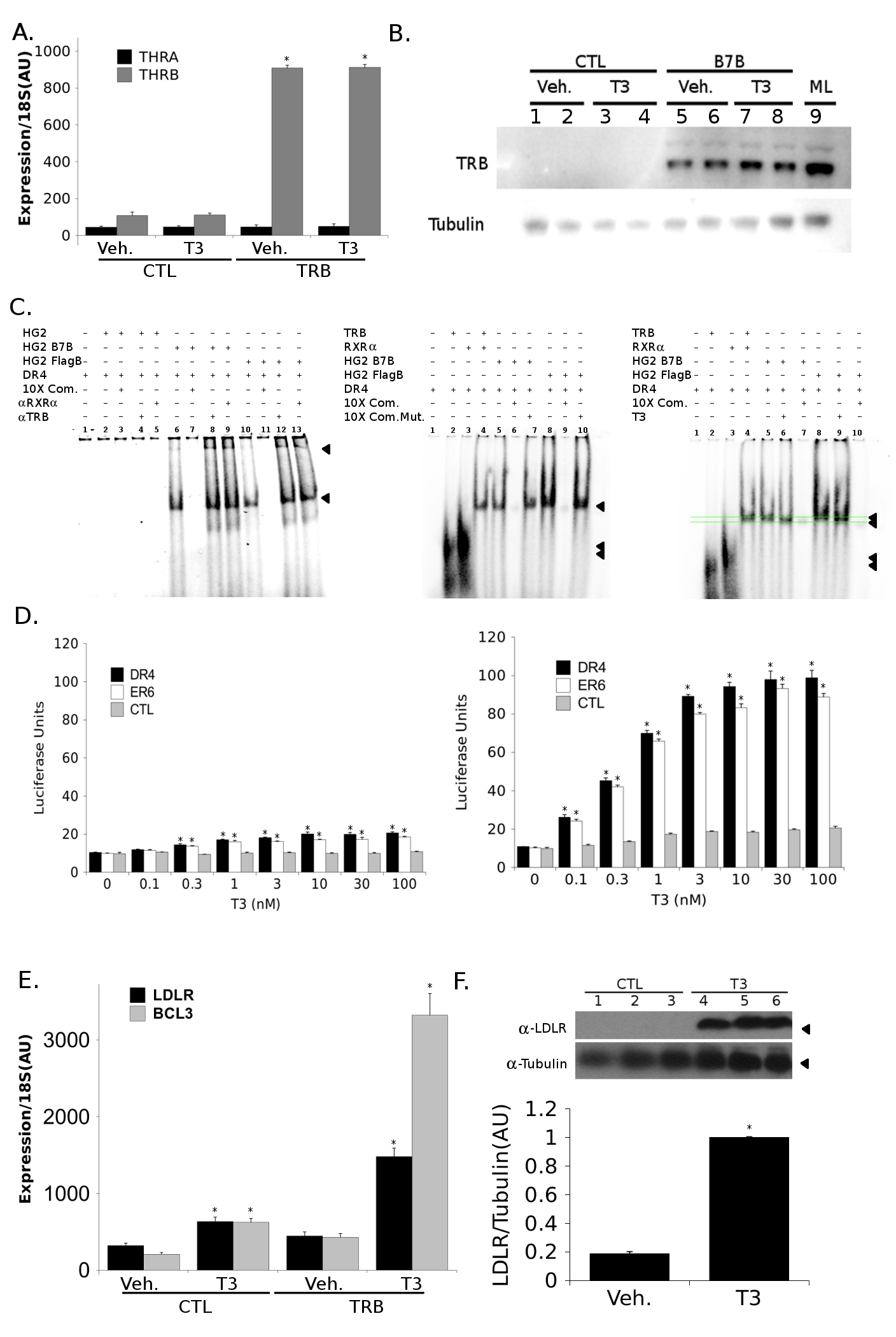

Supplement: Figure S1 — BioChIP-modified TRβ constructs exhibit similar activity to endogenous TRβ. A. BLRP-tagged TRβ was expressed in HepG2 cells at a level 8–10 fold higher than that endogenously expressed in parental HepG2 cells, as assessed by realtime PCR, normalized to 18S RNA. B. TRβ protein levels in B7B cells (lanes 5–8, 20 µg cellular protein per lane) were found to be substantially less than that expressed in mouse liver (lane 9, 20 µg protein per lane) as assessed by western blot. C. Modes of DNA binding were assessed by EMSA assays, as indicated. B7B cells elicited mobility shift of a DR4 element (left panel, lanes 6–9), similar to that of previously characterized [54] Flag-tagged TRβ cell lysates (“HG2 FlagB”, lanes 10–13). Supershift of complexes with RXRα or TRβ antibodies indicated the predominantly heterodimeric composition of complexes (left panel, upper band). Analysis of B7B cell lysates, in comparison to in vitro-expressed RXRα and TRβ protein alone (middle panel, lanes 2&3) or combined lysates (lane 4) confirmed a predominantly heterodimeric mode of binding to DR4 elements (lanes 5–7), similar to Flag TRβ lysates (lanes 8–10). Analysis of T3-treated cell lysates revealed a characteristic change in mobility, as compared with untreated lysates, similar in B7B (right panel, lanes 5–7) and HG2 FlagB lysates (lanes 8–10). D. Transcriptional activation of DR4 and ER6 elements were quantified in comparison to controls in luciferase reporter assays in HepG2 cells (left panel) or TRβ-expressing HepG2 cells (right panel). E. Expression of defined TRβ target genes was quantified by realtime PCR of target genes in indicated cell models, normalized to 18S RNA. F. Protein expression of the TRβ-regulated gene LDLR was assessed by western blot in TRβ-expressing HepG2 cells, showing substantially increased protein levels after T3 activation of TRβ. (TIF) [file pone.0081186.s001.tif]

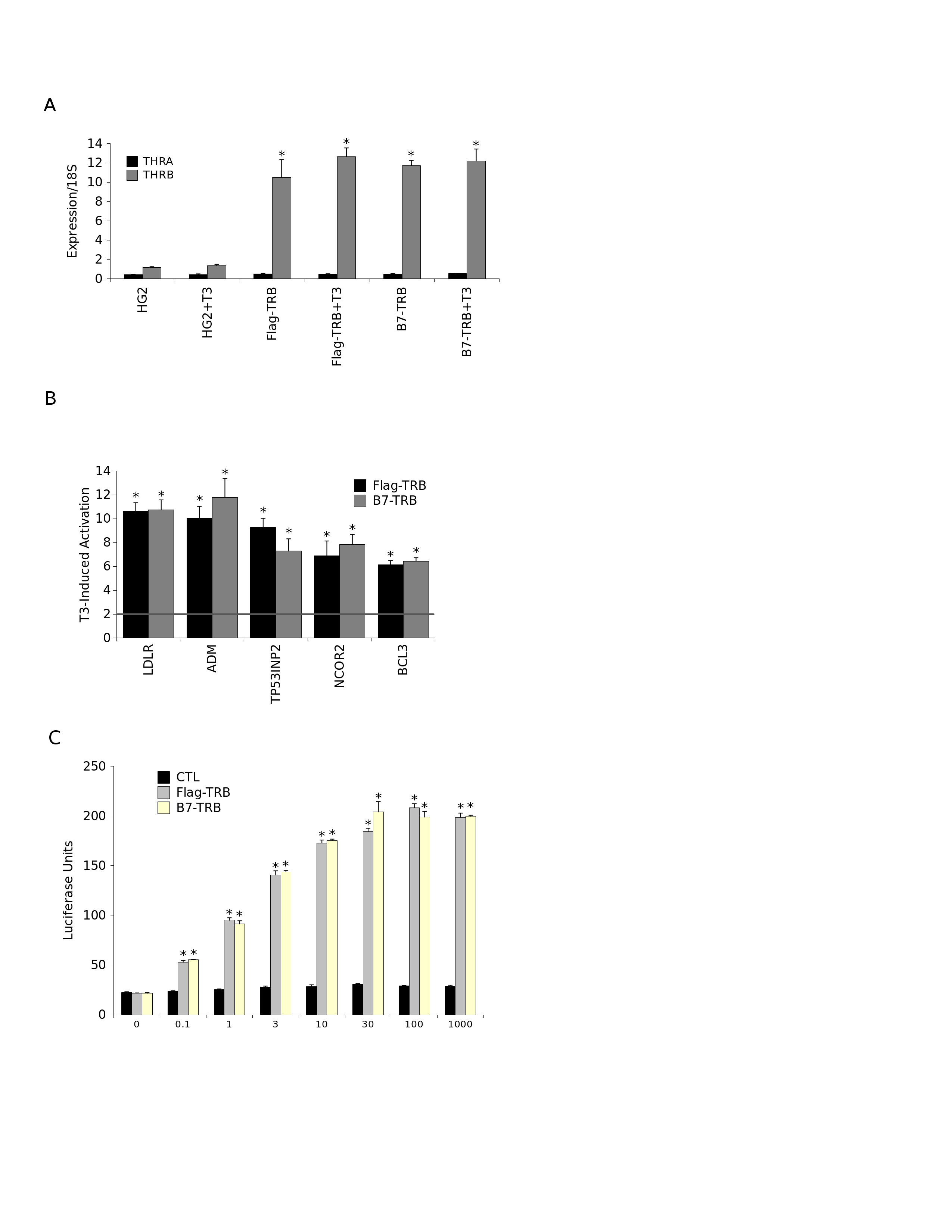

Supplement: Figure S2 — Comparison of B7B cells and HepG2-TRβ (Flag) cells. A. Expression of BLRP-tagged TRβ or Flag-tagged TRβ was 8–10 fold higher than endogenous TRβ expression in HepG2 parental cells and was not affected by treatment with 100 nM T3 for 8 hours, as assessed by realtime PCR, normalized to 18S RNA.Expression of TRα was similar in all samples. B. Expression of defined TRβ target genes was induced with 8 hour T3 treatment to a similar extent in BLRP-tagged TRβ and Flag-tagged TRβ cells, as assessed by realtime PCR of indicated targets, normalized to 18S RNA. C. A DR4 luciferase reporter construct showed a similar transcriptional activation profile after treatment of BLRP-tagged TRβ and Flag-tagged TRβ cells; little activation was observed in HepG2 parental cells. (TIF) [file pone.0081186.s002.tif]

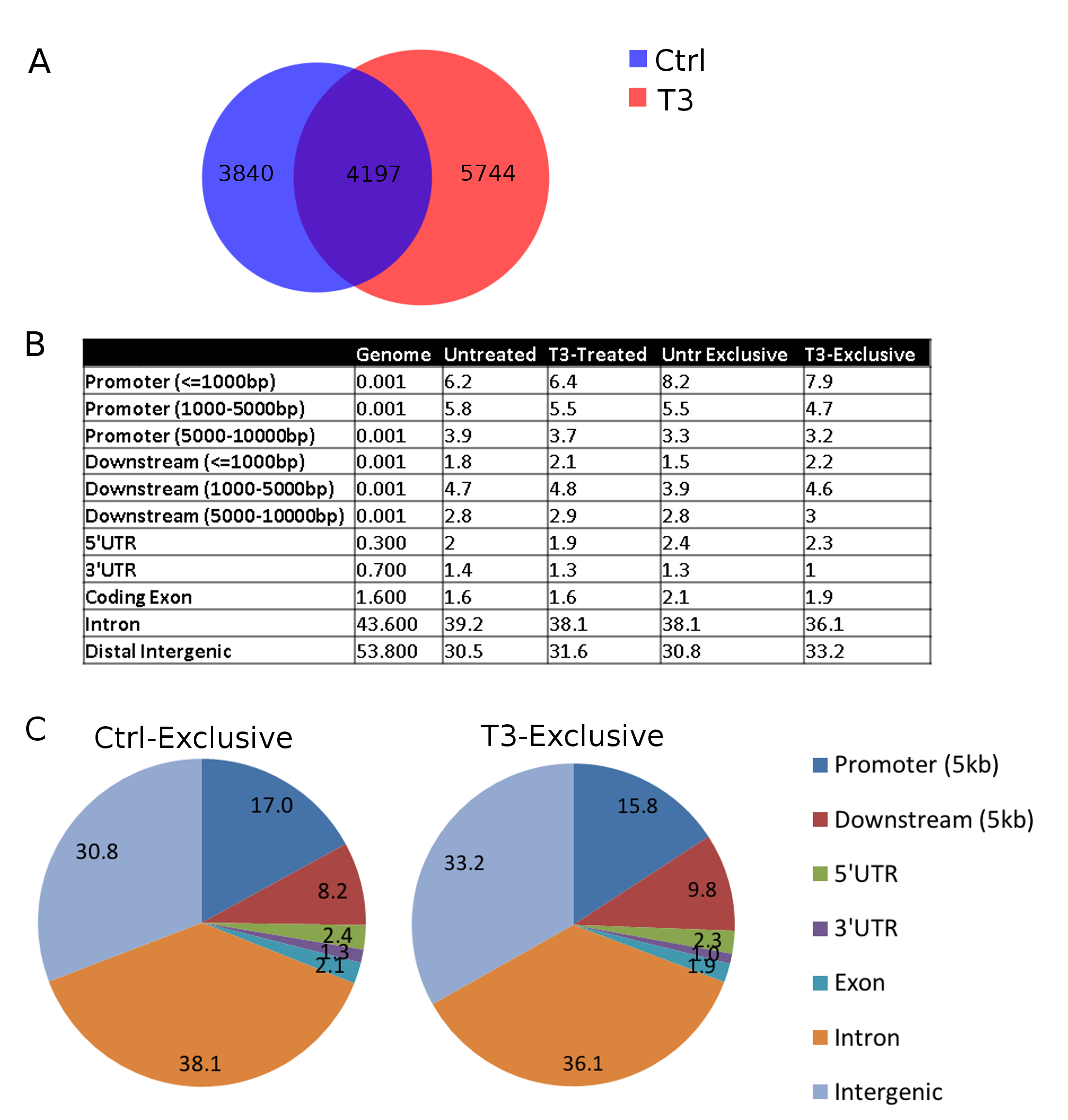

Supplement: Figure S3 — Summary of binding events in Trβ-expressing cells. A. TRβ binding peaks were mapped to genomic regions, using the methods described. Approximately 30% of binding to sites occurred only in the presence or absence of T3, as depicted in the Venn diagram shown. B. Percentages of total binding events within specific regions, as assessed with the CEAS analysis program [68], [69], are shown, including those occurring exclusively in untreated samples, or exclusively in T3-treated samples are indicated. C. Percentages of binding events, which were exclusively observed in untreated or T3-treated samples, was mapped to the genomic regions indicated, using the CEAS analysis program, and depicted in the charts as labeled. (TIF) [file pone.0081186.s003.tif]

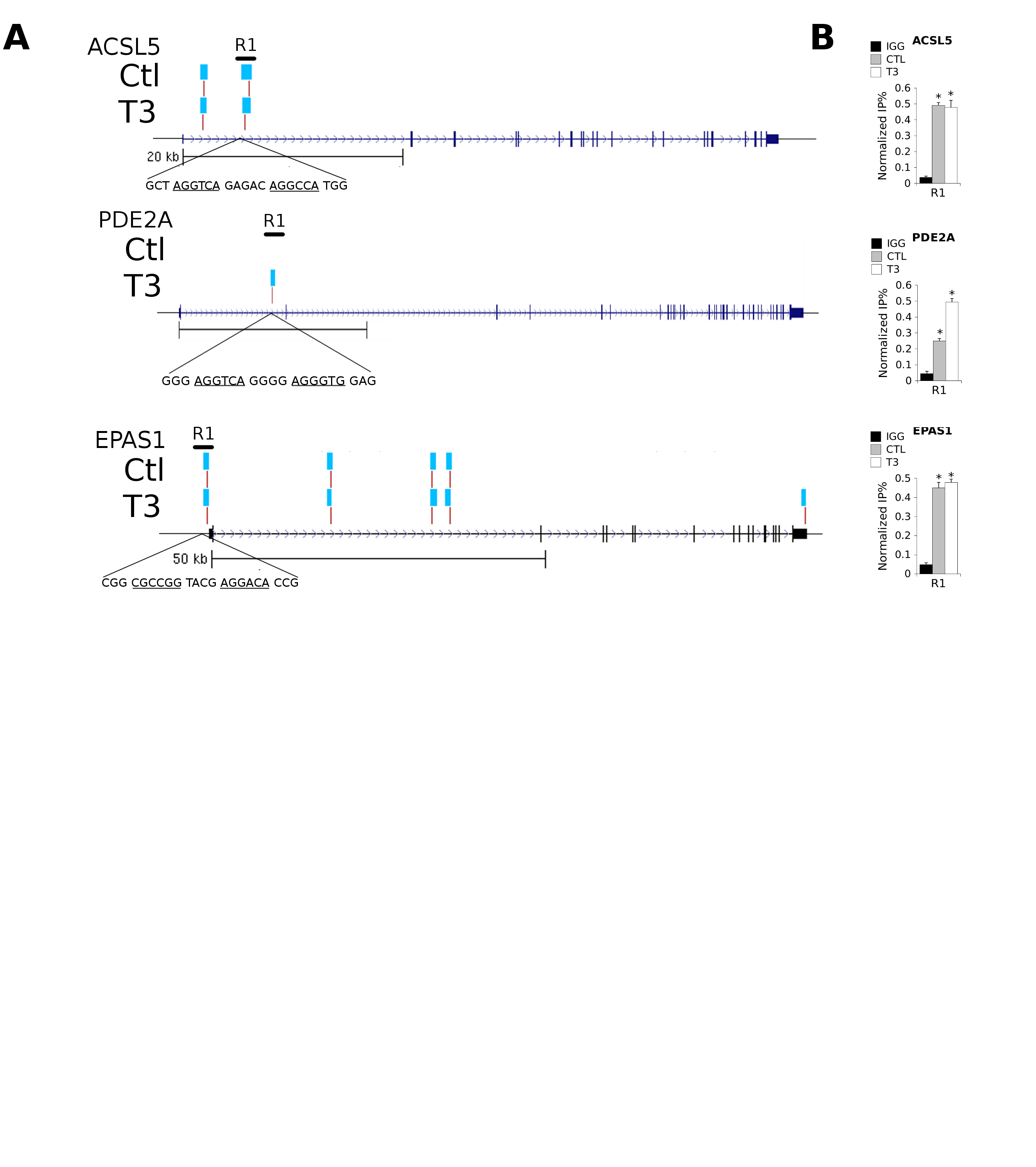

Supplement: Figure S4 — Characterization of TRβ binding near induced genes. A. Patterns of TRβ binding were depicted at individual target gene loci, represented using the same format as Fig. 4. Observed binding patterns included 5′, 3′ and intronic binding events, as shown in genomic data tracks (UCSC Genome Browser). B. Patterns of TRβ binding at the regions indicated with ChIP, analyzed by QPCR. (*P<0.05 by Student's T-Test). (TIF) [file pone.0081186.s004.tif]
