# Supplementary material for: Genome-Wide Binding Patterns of Thyroid Hormone Receptor Beta
Source: PLoS One. 2014 Feb 18;9(2):e81186. doi: 10.1371/journal.pone.0081186 (PMC3928038; doi:10.1371/journal.pone.0081186)
Supplement: Table S1 — Response elements characterized in reported data. Hormone response elements are listed by their associated genes (Column 1). The DNA sequence of each element is provided, with spaces to indicate half-site position (Column 2), and the assays used to evaluate the elements described (Column 3). (PDF) [file pone.0081186.s005.pdf]

Table S1. Response elements characterized in reported data.

Gene	Sequence	Assay
LDLR	TGG AGGTCA CCGCG GGCTCA GGG	ChIP
BCL3-R1	ATA GGGTAA GGGTTA ACGCCA GCA	ChIP
BCL3-R2	TAC AGGCCA TGGT GCTGAG TAA	ChIP
BCL3-R3	CTT GGAGTT TCTT AGGGCA GT	ChIP
NCOR2-R1	ACA GGACGT TGGG AGGCCA ACG	ChIP
NCOR2-R2	GCA TGACCC TCTGGA CTGGCA AAC	ChIP
ADSSL1	CAG TGACGC CCCGG TGACCT CCG	ChIP
ADSSL2	CAC TGGCCC TG CGGTCA TTG	ChIP
ADSSL3	GGG TGACCT TTTG TGCTCA CCA	ChIP
SOX7-R1	CAG GGGAGA CTCA GGGTCA TAG	ChIP
ADM TRE-1	GGC TGGGGA GGCTCC TGGTCA CCG	EMSA
ADM TRE-2	CCAC CGGGCA GCCC AGGCCC CGC	EMSA
INT-1	AA AGGTCA GGGG ATATTG TG	ChIP
INT-2	TC CCAGCA AGAACA CAGTCA GG	ChIP
INT-3	CA AGTTCA CTGGG CGGTCA CA	ChIP
DR4	GG AGGTCA TGTG AGGTCA GG	EMSA, Transactivation Assay
ER6	GG TGACCT TGTGTG AGGTCA GG	Transactivation Assay
