# Supplementary material for: Genome-Wide Binding Patterns of Thyroid Hormone Receptor Beta
Source: PLoS One. 2014 Feb 18;9(2):e81186. doi: 10.1371/journal.pone.0081186 (PMC3928038; doi:10.1371/journal.pone.0081186)
Supplement: Table S2 — Primers used for chromatin immunoprecipitation. TRß binding at indicated peaks was assessed by realtime PCR of chromatin IP-enriched samples with indicated primer sets. Primer sets were designed to specifically amplify indicated genomic regions. (DOC) [file pone.0081186.s006.doc]

**Table S2. Primers used for chromatin immunoprecipitation**

| Peak | Location | Forward Primer | Reverse Primer | | |
| --- | --- | --- | --- | --- | --- |
| LDLR R1 | chr19:11199987 | GAGCTTCACGGGTTAAAAAGCCGATGTCAC | TGTGAGGTTTCTAGCAGGGGGAGGAGTTTG | | |
| BCL3 R1 | chr19:45246015 | GAAGAGTTCCAGGGTGCGAA | CTGGGAGAAGTGGCCATGAG |  |  |
| BCL3 R2 | chr19:45268567 | AAGACCCACATCTCAAGGCG | TTCTCAGCCACCGTGTCTTC |  |  |
| BCL3 R3 | chr19:45278019 | AGCCACAGCTCTGAAAGAGG | ACTGGCAAGTATTGGCAAGGA |  |  |
| NCOR2 R1 | chr12:125012627 | ACGACTGCCATAAACAGGCA | CTTCCAGAGAGCCTTGGTGG |  |  |
| NCOR2 R2 | chr12:124859307 | GGGTGTCCTTGCCCTAAGTC | CCATCCCTGAGGTCAACACC |  |  |
| ADSSL1 R1 | chr14:105190840 | TGAGTTGGGGACACCCGAT | AAGTTGACCCCTTTGTGCCT |  |  |
| ADSSL1 R2 | chr14:105198190 | ATATGTGCCGAGGCAAGGAC | ATTCTGGGTTTGGGGGTCAC |  |  |
| ADSSL1 R3 | chr14:105202088 | GACTCACCCAAGACCATCCC | CCTGCTGTGAACTAGGGACC |  |  |
| SOX7 R1 | chr8:10571463 | CCACAAGGTTGGGCTCAGAT | CACCATCTCCATCCTGCCTC |  |  |
| ADM R1 | chr11:10324896 | GTCCGCTCAGGTGACTCCTTCCAGG | CGAGTCCCCATCTTAGAGCCGGTCC | |  |
| ADM R2 | chr11:10325685 | TTCCTTGCCTGACTCAAGGGTGGCTGTGAAGCTC | TGACTGCCAGGGAGCACACTAACGCTTCACG | | |
| ADM R3 | chr11:10326235 | TGTCCCTTCCGCGGGCTCTTGCTGTTCTTC | AGGCGGTTGGTGCAGGAACTGCCACAGCC | | |
| ADM R4 | chr11:10329132 | GTGTCCTGGGTGCGAATCAGGGCTT | GAGGTGTCCCCACGCATTGCTAGGG | |  |
| ADM R4 | chr11:10329390 | CCTAGCAATGCGTGGGGACACCTCC | GGCTGTGTGCTGACTCCCACGTAGG | |  |
| EPAS1 R1 | chr2:46523926 | TTGCACTGCTTCTCCCAGAC | GCCAGGTGGGGATAATTTCT |  |  |
| ACSL5 R1 | chr10:114133874 | ACTCTCAGGACAGTACACAGTAGCTTCGGG | AATCCCACTTTCTTTCCCCACACCAATCCC | | |
| PDE2A R1 | chr11:72343293 | CCACTCACCCAGTCATGTCC | TTCTCCAGTGAAACTGGCCC |  |  |
| INT-1 | chr1:79698610 | CCAGCCCAGTCTTTTTGCTT | TGATGTCAAGAGGCAGAACACA |  |  |
| INT-2 | chr21:46782324 | GAAGCAACCTGACCACACCA | AGGCAGGGCTGTGTCCTC |  |  |
| INT-3 | chr21:44763130 | GCCTTCAGAGGGCAGAACC | CGCCAGACGGAAGGTAAAC |  |  |
