# Supplementary material for: Genome-Wide Binding Patterns of Thyroid Hormone Receptor Beta
Source: PLoS One. 2014 Feb 18;9(2):e81186. doi: 10.1371/journal.pone.0081186 (PMC3928038; doi:10.1371/journal.pone.0081186)
Supplement: Table S3 — Perl script used for analysis of bound sequences. Sequence composition of TRß-binding genomic regions was analyzed with the included Perl script, confirmed to run on Ubuntu Standard Distribution 11.1, in addition to the analysis procedures described in text. This script analyzes two BED-formatted input files by evaluating the length-normalized bidirectional site occurrences. (PDF) [file pone.0081186.s007.pdf]

```perl
#!/usr/bin/perl
#This script will analyze multiple sequences in input file for occurance
of
#regular expressions, provided in DATA section.
#run command: perl scanseq.pl
#Input file format: multiple sequence fasta format
#Output file formats:
#     multiple sequence fasta format (for each regex)
#     tab-delimited text file (summary)

#Read in regular expressions:
@regexlines =<DATA>;

#Initialize sequence files:
foreach $promofile ("TRB_Untr.fasta","TRB_1hr.fasta"){
#foreach $promofile ("TestSet.fasta"){
$promocount = 1;
$basespace = 0;
$totalhits = $promohits = $promoters = 0;
$sumfile = "$promofile.tab";
open(SUM,">>$sumfile") or die "Error writing log file $logfile: $!\n";

#Evaluate sequence file format; end if no valid lines found:
open(PRO,"<$promofile")or die "Error reading input file: $promofile $!\n";
while(!eof(PRO)){
  $line = <PRO>;
  if($line =~ /^>/){
    $promoters++;
  }
}
close(PRO);
if ($promoters < 1){die "Check format of promo file: $promofile\n";}
print "Searching $promoters sequences:\n";

#Analyze all sequences found in file for regex:
print "Analysis of sequences in: $promofile\n";
print "Searching $promoters sequences in $promofile\n";
print "Regex\t\tMotif\t% sequences\t\tEnrichment\n";
print SUM "Search of $promoters sequences in $promofile for regex:\n";
print SUM "Regex\tMotif\t% sequences\tEnrichment\n";
foreach $regexline (@regexlines){
  chomp($regexline);
  $basespace= $promohits = $totalhits = $pospromo = 0;
  ($regexname,$regex,$nrl,$pal)=split(/\t/,$regexline);
  print "$regexname\t$regex\t";
  print SUM "$regexname\t$regex\t";
  open(PRO,"<$promofile")or die "Error opening promo file $promofile: $!
\n";
  $header = $nextheader = <PRO>;
  chomp($header);
  $outfile = "SSO_$promofile._$regexname.out";
  open(OUT,">$outfile") or print "Error opening output file $outfile:$!
```

```
\n";
  for($i =0; $i<$promoters;$i++){
    $header = $nextheader;
    chomp($header);
    $promohits = $seq = $line = 0;
    while($line !~ /^>/ && !eof){
      $seq = $seq.$line;
      $line = <PRO>;
      chomp($line);
    }
    $nextheader = $line;
    while ($seq =~ m/\s/){
      $seq =~ s/\s//;
    }
    while($seq =~ m/()($regex)()/gi){
      $promohits++;
      $totalhits++;
      print OUT "$header $regexname $promohits $totalhits\n";
      print OUT "$1 $2 $3\n";
    }
    if($pal>0){
      $basespace += (length($seq)-2*($nrl-1));
    }
    else{#search reverse sequence for non-palindromic regex
      $basespace += 2*(length($seq)-2*($nrl-1));
      $seq = reverse($seq);
      $seq =~ tr/A,G,C,T,N,a,g,c,t,n/T,C,G,A,N,t,c,g,a,n/;
      while($seq =~ m/()($regex)()/gi){
        $promohits++;
        $totalhits++;
        print OUT "$header $regexname (reverse) $promohits $totalhits\n";
        print OUT "$1 $2 $3\n";
      }
    }
    if($promohits>0){
      $pospromo++;
    }
  }
  $perpromo = $totalhits/$promoters;
  $pospromopercent = 100*$pospromo/$promoters;
  $expected = $basespace/(4**$nrl);
  $escore = $totalhits/$expected;
  print "$pospromopercent\t$escore\n";
  print SUM "$pospromopercent\t$escore\n";
  close(PRO);
  close(OUT);
}
$totalhits = $promohits = 0;
close(SUM);
}
```

```
#Regular expressions in format:
#name\t regex\t length
__DATA__
Halfsite  AGGTCA  6 0
HalfsiteN AGG.CA  5 0
DR4-N AGG.CA....AGG.CA  10  0
DR4-3 AGG.......AGG.CA  8 0
ER6 ...CCT......AGG.CA  8 1
IP0 ...CCTAGG.CA  8 1
IP1 ...CCT.AGG.CA 8 1
Neg1  TTTGGG  6 0
Neg2  CCCCTCAGGCGC  12  0
```
