# Supplementary material for: Genome-Wide Binding Patterns of Thyroid Hormone Receptor Beta
Source: PLoS One. 2014 Feb 18;9(2):e81186. doi: 10.1371/journal.pone.0081186 (PMC3928038; doi:10.1371/journal.pone.0081186)
Supplement: Table S4 — Primers used for realtime PCR-based analysis of gene expression. Gene expression was assessed by realtime PCR with primer sets, designed to specifically assess expression of indicated genes. (PDF) [file pone.0081186.s008.pdf]

**Table S4. Primers used for chromatin immunoprecipitation**

| Gene | Forward Primer | Reverse Primer |
|---|---|---|
| *18S* | TCAATCTCGGGTGGCTGAACG | GGACCAGAGCGAAAGCATTTG |
| *THRA* | AGGTCACCAGATGGAAAGCG | AGTGATAACCAGTTGCCTTGTC |
| *THRB* | GGACAAGCACCCATCGTGAAT | CTCTGGTAATTGCTGGTGTGAT |
| *LDLR* | CGACAGATGCGAAAGAAACGA | CCCGGATTTGCAGGTGACA |
| *BCL3* | CCGGAGGCGCTTTACTACC | TAGGGGTGTAGGCAGGTTCAC |
| *NCOR2* | TGCAGATCATCTACGACGAGA | TCCGCATCGCCTGGTTTATTT |
| *ADSSL1* | CGGACGCCGACATCATCAG | CCCGTTGCCAATGAAGGACA |
| *SOX7* | AGCCGGAGCAGACCTTCTT | GCCGGGGAGTAATAGGCAG |
| *ADM* | ATGAAGCTGGTTTCCGTCG | GCCCACTTATTCCACTTCTTTCG |
