# Supplementary material for: Genome-Wide Binding Patterns of Thyroid Hormone Receptor Beta
Source: PLoS One. 2014 Feb 18;9(2):e81186. doi: 10.1371/journal.pone.0081186 (PMC3928038; doi:10.1371/journal.pone.0081186)
Supplement: Table S5 — Peaks. Peaks of TRβ binding are listed for each sample by start and end position. Genes proximal to peaks are listed by name, strand and distance from peak. Peaks over-represented in TR-enriched samples, relative to unenriched control DNA were assessed using the QuEST package, version 2.4. (DOC) [file pone.0081186.s009.doc]

**Table S5.** **Negatively regulated genes and associated regulatory elements**

| Gene | Fold Inhibition | Position | Element | Sequence |  |  |
| --- | --- | --- | --- | --- | --- | --- |
| PDE2A | 4.35 | chr11:72,343,393 | TRE R-3-F | CTG TGCCCT GCA GGGTCT TCC | | |
|  |  | chr11:72,344,083 | TRE F-3-F | CAT AGGCCC CCC AGGCCA GCT | | |
|  |  | chr11:72,396,164 | CTCF | AGA TATATTTATATT ATC | |  |
|  |  | chr11:72,301,407 | CTCF | GTC CCTCAGGCGC CGC | |  |
| ALDH3A1 | 2.08 | chr17:19,656,206 | TRE F-1-F | CTT GGGTCA G AGAAGC TGC | | |
|  |  | chr17:19,656,240 | TRE F-3-F | CGG AGGTGA GTG CGAGCA AGG | | |
|  |  | chr17:19,657,564 | CTCF | TAT ATTTTGTTATAT AAA | |  |
| NFE2 | 1.96 | chr12:54,697,539 | TRE F-0-F | TTG AGGTCA TCTACT ACT | | |
| SOX9 | 1.81 | chr17:70,122,536 | TRE F-4-F | CAG AGAAAC GTTC AGGTCA TTT | | |
|  |  | chr17:70,123,296 | CTCF | CAT TTTTCATTAAAT AGA | |  |
| C9ORF169 | 1.72 | chr9:140,119,375 | TRE R-1-F | CCG TGACCT C AGGCTC ACC | | |
|  |  | chr9:140,122,322 | TRE F-4-F | GAG ATGTGA GCAG AGGCCA AGG | | |
|  |  | chr9:140,119,410 | NTRE | ACC TCAGGC TCA | |  |
|  |  | chr9:140,119,680 | NTRE | AAC TCAGGC ACT | |  |
|  |  | chr9:140,121,344 | CTCF | GGA AATATCTAATT CCT | |  |
| SYNC | 1.75 | chr1:33,169,116 | TRE F-4-F | TGA GGGTCA AACC AGCACT GAC | | |
|  |  | chr1:33,169,800 | TRE F-4-F | TGC TGCCCG CCAG AGGAGA GCT | | |
